# Elevated plasma S100A1 level is a risk factor for ST-segment elevation myocardial infarction and associated with post-infarction cardiac function

**DOI:** 10.7150/ijms.35037

**Published:** 2019-08-06

**Authors:** Linlin Fan, Baoxin Liu, Rong Guo, Jiachen Luo, Hongqiang Li, Zhiqiang Li, Weigang Xu

**Affiliations:** 1 Institute of Biomedical Sciences, Department of Cardiology, Shanghai Institute of Cardiovascular Disease, Fudan University, Shanghai, 200032, China;; 2 Department of Cardiology, Shanghai Tenth People's Hospital, Tongji University School of Medicine, Shanghai, 200072, China;; 3 Community Health Service Center of Pengpu New Estate, Jing'an District, Shanghai, 200435, China

**Keywords:** ST-segment elevation myocardial infarction, S100A1, Cardiac function, Biomarker, Cardiovascular disease.

## Abstract

Aim: To investigate the association between plasma S100A1 level and ST-segment elevation myocardial infarction (STEMI) and potential significance of S100A1 in post-infarction cardiac function. Methods: We examined the plasma S100A1 level in 207 STEMI patients (STEMI group) and 217 clinically healthy subjects for routine physical examination without a history of coronary artery disease (Control group). Baseline characteristics and concentrations of relevant biomarkers were compared. The relationship between S100A1 and other plasma biomarkers was detected using correlation analysis. The predictive role of S100A1 on occurrence of STEMI was then assessed using multivariate ordinal regression model analysis after adjusting for other covariates. Results: The plasma S100A1 level was found to be significantly higher (P<0.001) in STEMI group (3197.7±1576.0 pg/mL) than in Control (1423.5±1315.5 pg/mL) group. Furthermore, the correlation analysis demonstrated plasma S100A1 level was significantly associated correlated with hypersensitive cardiac troponin T (hs-cTnT) (r = 0.32; P < 0.001), creatine kinase MB (CK-MB) (r = 0.42, P < 0.001), left ventricular eject fraction (LVEF) (r = -0.12, P = 0.01), N-terminal prohormone of brain natriuretic peptide (NT-proBNP) (r = 0.61; P < 0.001) and hypersensitive C reactive protein (hs-CRP) (r = 0.38; P < 0.001). Moreover, the enrolled subjects who with a S100A1 concentration ≤ 1965.9 pg/mL presented significantly better cardiac function than the rest population. Multivariate Logistic regression analysis revealed that S100A1 was an independent predictor for STEMI patients (OR: 0.671, 95% CI 0.500-0.891, P<0.001). In addition, higher S100A1 concentration (> 1965.9 pg/mL) significantly increased the risk of STEMI as compared with the lower level (OR: 6.925; 95% CI: 4.15-11.375; P<0.001). Conclusion: These results indicated that the elevated plasma S100A1 level is an important predictor of STEMI in combination with several biomarkers and also potentially reflects the cardiac function following the acute coronary ischemia.

## Introduction

Cardiovascular disease (CVD) has caused increasing morbidity and mortality and been regarded as a global substantial health-care burden in recent decades 1. As the most serious type of coronary heart disease, ST segment elevation myocardial infarction (STEMI) still caused high case fatality and poor prognosis 2. This is partly due to the delay in diagnosis and lack of highly sensitive and specific markers 3. Although the Fourth Universal Definition of Myocardial Infarction and the STEMI guidelines have issued elevated serum cardiac troponin (cTn) as the essential biomarker 4, 5, the clinical applications of cTn still have certain limitations. The rise of cTn occurs 3-4 hours following the onset of myocardial injury, which may not be efficient in early diagnosis of STEMI within first 1-2 hours. With the development of high-sensitivity cTn (hs-cTn) analysis, diagnostic sensitivity has been further improved, however, specificity is relatively reduced since serum cTn levels were also increased in renal failure or pulmonary embolism patients without MI 6, 7. Other myocardial necrosis biomarkers, such as creatine kinase MB (CK-MB) and myoglobin (MYO), were similarly lacked cardiac specificity for diagnosing myocardial infarction to some extent 8, 9. These limitations and urgent clinical requirements have promoted identifications of novel biomarkers for MI, including neuroendocrine, inflammatory, genetic and molecular biomarkers. For instance, N-terminal B-type natriuretic peptide (NT-proBNP) was closely associated with left ventricular function and 1-year survival in MI patients 8. Several studies have also reported C-reactive protein (CRP) was a diagnostic biomarker for AMI and could potentially reflect the extent of myocardial injury in STEMI 8, 10. Several cardiac-specific microRNAs have been proved to play important roles in MI 11. More recently, several leukocyte-derived microvesicles were found to constitute important elements in pathogenesis of STEMI 12, 13. Due to development and application of new biomarkers in MI, it seemed that a single biomarker could not possibly provide sufficient sensitivity and specificity in diagnosis and prognosis of MI. A multibiomarker approach may enhance the early diagnostic value and provide more information for the early risk stratification of AMI. Here, we reported a new molecular biomarker, S100A1, which may possibly play important roles in STEMI.

S100A1 is the most abundant member from S100 proteins which are a large family of EF-hand Ca^2+^-binding proteins that are characterized by tissue and cell-specific expressions in vertebrates 14, 15. S100A1 is highly expressed in heart and skeletal muscle and at low levels in most normal tissues 16. It could regulate Ca^2+^ homeostasis via interaction with regulatory proteins such as SERCA2a, ryanodine receptors, L-type calcium channels and Na^+^/Ca^2+^ exchangers. Thus, S100A1 is involved in a variety of intracellular activities such as muscle contractility, cell differentiation and gene expression 17, 18. Moreover, S100A1 protein can be also secreted from cells and act as an extracellular chemotactic cytokine that is related to inflammation 19.

S100A1 has recently emerged as an attractive target in CVD as cardiac contractility dysfunction and inflammatory response are generally the basis of cardiac injury 20, 21. Since S100A1 plays a crucial role in these processes, decreased S100A1 expression in cardiomyocytes has been well documented in heart failure 22-24. However, few studies have focused on the diagnostic performance of circulating S100A1 levels in patients with STEMI. In this retrospective study we investigated the diagnostic value of S100A1 in detection of STEMI.

## Methods

### Study population

The study population was constituted by two groups: From February 2013 to December 2015, a total of 270 STEMI patients who had undergone primary percutaneous coronary intervention (PCI) at the Catheterization Laboratory of Department of Cardiology, Shanghai Tenth People's Hospital, were enrolled into STEMI group. STEMI was diagnosed in compliance with the criteria issued by the ACC and ESC 25: typical elevated and gradual fall cTnT concentration above the 99th percentile of the upper reference limit (hs-cTnT ≥ 0.014 ng/mL), with an acute onset of typical ischemic angina, or surface ECG showing: ST-segment elevation (≥ 0.2 mV in men or ≥ 0.15 mV in women in leads V2-V3 and/or ≥ 0.1 mV in other leads). Population in healthy control group were 217 clinically healthy subjects for routine physical examination in outpatient department. All subjects in these two groups were comparable for age and gender, respectively. The exclusion criteria included 1) autoimmune, malignant or infectious diseases or diseases of the connective tissue, 2) severe hepatic or renal failure, 3) severe valvular or congenital heart disease, and 4) acute cerebrovascular accident.

After admission, clinical data were collected and documented for all patients, including sex, age, presence of hypertension, tobacco use, and diabetes; biochemical tests to determine the levels of blood glucose, total cholesterol (TC), triglycerides (TG), high density lipoprotein (HDL), low density lipoprotein (LDL), high sensitive cardiac troponin T (hs-cTnT), creatine kinase MB (CK-MB) and high-sensitivity C-reactive protein (hs-CRP) were performed. Findings from standard 12-lead ECGs, echocardiography, and coronary angiography (CAG) were also collected. This study complies with World Medical Association's Declaration of Helsinki and was approved by the Ethics Committee of Shanghai tenth people's hospital. All patients recruited in the current study provided written informed consent.

### Doppler echocardiography

Echocardiographic examinations were performed at rest, with the patient semirecumbent in the left lateral position. All scans were performed and reported by cardiologists with advanced training in echocardiography, using a GE Vivid 7 (GE Healthcare, Piscataway, NJ, USA) ultrasound machine with a M4S (1.7-3.4 MHz) transducer. Left ventricular measurements were analyzed using the M-mode from the parasternal long axis according to American Society of Echocardiography guidelines 26. Left ventricular mass (LVM) and ejection fraction (LVEF) were also calculated from M-mode measurements 27, 28. The pulsed Doppler sampling volume was placed between the tips of the mitral valve leaflets to obtain maximum filling velocities in passive end-expiration by using a 3-5 mm sample volume. A standardized loop of 10 cardiac cycles was downloaded to the computer for analysis of the peak of early diastolic velocities (peak E), the peak of late diastolic velocities (peak A), the deceleration time of the peak E velocity (DT), and isovolumic relaxation time (IVRT). Pulsed wave Doppler tissue imaging (DTI) was acquired in the apical 4-chamber view placed over the myocardium, on the septum, at the level of the mitral annulus. Systolic motion (s' wave) and early (e') and late diastolic (a') mitral annulus velocities were obtained. The e' wave velocities from the septal and lateral walls were averaged and the ratio of the transmitral E wave to the average e' velocity (E/e' ratio) was calculated as an indicator of left ventricular filling pressure.

### Blood sample

Fasting venous blood samples were obtained at admission to the Catheterization Laboratory for emergency reperfusion therapy (STEMI group) or in the next morning after at least 4 h of fat fasting and before 10 a.m. (Control group). Approximately 5 mL sample was placed in an EDTA tube and centrifuged at 3000 rpm for 10 min. The plasma was separated at 4 °C for analysis.

### S100A1 assay

The concentration of the serum calcium-binding protein S100A1 was measured using an enzyme-linked immunosorbent assay (ELISA) kit from Lifespan BioScience (WA, USA) and followed the manufacturer's instructions. The ELISA kit has a sensitivity less than 0.061 ng/ml. The intra-assay and inter-assay CVs were <10% and <12%, respectively, and the detection range for the kit was 0.156-10 ng/mL. Samples from the same patient were assessed in the same plate at the same time and using an internal control sample assessed in duplicate to validate our results. The experiment was repeated at least three times.

### Measurements of other biomarkers

Apart from S100A1, all the other biochemical markers were measured and analyzed using specific reagents and instruments in Department of Clinical Laboratory Medicine, Shanghai Tenth People's Hospital. The serum hs-CRP was detected by immunonephelometric assay. The plasma lipid and lipoprotein, including TC, TG, HDL-C, LDL-C, were detected by enzyme-colorimetric method. Hs-cTnT, CK-MB, and NT-proBNP were measured using electrochemiluminescence immunoassay. Fasting blood glucose (FBG) was determined using hexokinase method, and hemoglobin A1c (HbA1c) level was assessed by high-performance liquid chromatography method. The finally gathered results were carefully reviewed and reported by professionals.

### Definitions

Hypertension was defined when systolic blood pressure/diastolic blood pressure ≥ 140/90 mmHg in the supine position, or use of antihypertensive drugs. Diabetes mellitus was identified by a fasting plasma glucose ≥ 7.0 mmol/L, or random plasma glucose ≥ 11.1 mmol/L, or if patients received insulin or oral medications for diabetes. Smoking history was defined by using ≥ 1 pack (20 cigarettes) per day at least 1 year, either at admission or in the past.

### Statistical analysis

SPSS 17.0 software (SPSS Inc., Chicago, IL, USA) was used for statistical analysis. Continuous variables are expressed as the mean ± standard deviation, and categorical variables as a percentage. Differences between groups were determined using t-Student test for independent samples with normal distribution and Mann-Whitney test for nonparametric samples. Linear regression was used for correlation analysis. The risks for STEMI were assessed in a logistic regression analysis. The difference was considered statistically significant at P < 0.05.

## Results

### Baseline Characteristics

**Figure 1:**
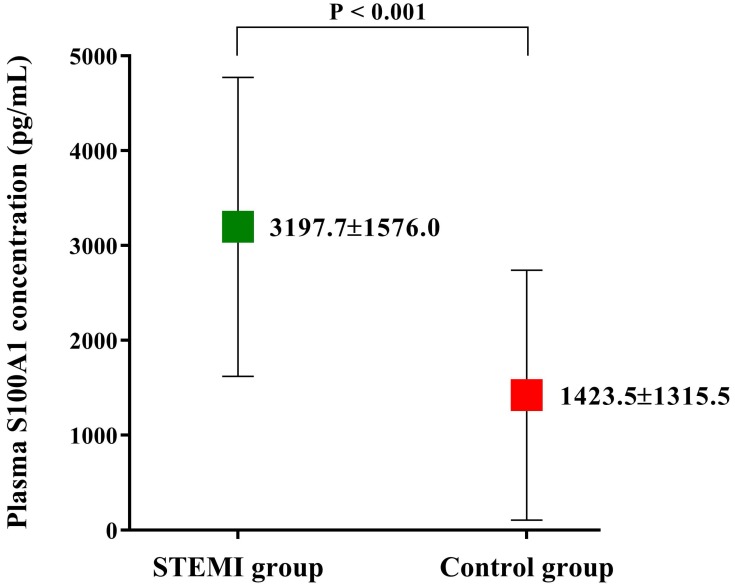
Plasma S100A1 level of the enrolled study population. Plasma S100A1 level was significantly higher in STEMI group than that in Control group. Abbreviations: STEMI, ST-segment elevation myocardial infarction.

Among the 604 patients inquired initially, 46 were not eligible and 42 not interested in the study. During the period of data collection, 12 patients were no longer interested and 17 patients with missing data. The biochemical indexes and clinical data of the finally enrolled 487 patients are shown in Table 1. The demographic characteristics such as age and gender were not significantly different among two groups. However, the incidence of diabetes mellitus was significantly higher in STEMI group than Control group (35.6% vs. 18.4%; P < 0.001). Patients in STEMI group also had significantly higher hs-cTnT, CK-MB, NT-proBNP and hs-CRP level levels (all p < 0.001, Table 1). The clinical data also showed a lower LVEF (58.3 ± 9.4% vs. 63.8 ± 10.3%; P < 0.001, Table 1) in STEMI group, which indicated patients in Control group may have a better cardiac function than in STEMI group. Plasma S100A1 level was found to be significantly higher in STEMI group than in Control group (3197.7±1576.0 pg/mL vs. 1423.5±1315.5 pg/mL; P < 0.001; Figure 1).

### S100A1 was significantly associated with cardiovascular risk factors and biomarkers

We detected the difference in plasma S100A1 levels between patients with and without cardiovascular risk factors, including gender, hypertension, diabetes mellitus, and smoking habit. The results were outlined in Table 2. Among all study population, 195 were with hypertension, 136 were with diabetes mellitus, and 206 had a smoking habit for at least one year. To clarify the relationship between S100A1 and age, we defined ≥ 65 years as old age according to the 2013 American College of Cardiology Foundation/American Heart Association Guideline for the management of patients with STEMI, which is also possibly a major risk factor of CAD 4. Our findings showed there existed statistical significance in S100A1 levels between patients with and without hypertension or smoking history, respectively (both P < 0.05; Table 2). Hs-CRP value <1, 1-3, and > 3mg/L were regarded as lower, average or higher relative risk factors in cardiovascular diseases 29. S100A1 level in patients with a hs-CRP value > 3 mg/L (2724.0 ± 1719.8 pg/mL) was significantly higher than in patients with a hs-CRP value 1-3 mg/L (1721.9 ± 1479.2 pg/mL) and < 1 mg/L (1206.7 ± 992.5 pg/mL) (both P < 0.001). S100A1 level in patients with a hs-CRP value < 1mg/L was relatively lower than patients with a hs-CRP value 1-3 mg/L, but the difference did not reach statistical significance (1206.7 ± 992.5 pg/mL vs. 1721.9 ± 1479.2 pg/mL; P = 0.108).

The correlation analysis demonstrated plasma S100A1 level was significantly correlated with hs-cTnT (r = 0.32; P < 0.001), CK-MB (r = 0.42; P < 0.001), NT-proBNP (r = 0.61, P < 0.001), and hs-CRP (r = 0.38, P < 0.001), respectively. In addition, the NT-proBNP level of STEMI patients and control subjects was significantly correlated with plasma S100A1 level, respectively (STEMI group: r = 0.42, P < 0.001; Control group: r = 0.47, P < 0.001). The significant correlation between S100A1 and hs-CRP was also observed in patients with an hs-CRP value > 3 mg/L (r = 0.12; P = 0.02) as well as 1-3 mg/L (r = 0.21; P = 0.04), respectively.

### Independent association between S100A1 and STEMI

Multivariate Logistic regression analysis was used to estimate the independent relationship between S100A1 and occurrence of STEMI after adjusting for other potential confounders (Table 3). Plasma S100A1 level was an independent risk factor in the occurrence of STEMI (OR: 0.671; 95%CI: 0.500-0.891; P < 0.001). In addition, diabetes (OR: 2.439; 95% CI: 1.597-3.731; P < 0.001), hs-cTnT (OR: 0.308; 95% CI: 0.209-0.455; P < 0.001), CK-MB (OR: 0.628; 95% CI: 0.536-0.701; P < 0.001), hs-CRP (OR: 0.338; 95% CI: 0.279-0.410; P < 0.001), and NT-proBNP (OR: 0.456; 95% CI: 0.275-0.756; P = 0.002) were significantly related to the incidence of STEMI.

**Figure 2:**
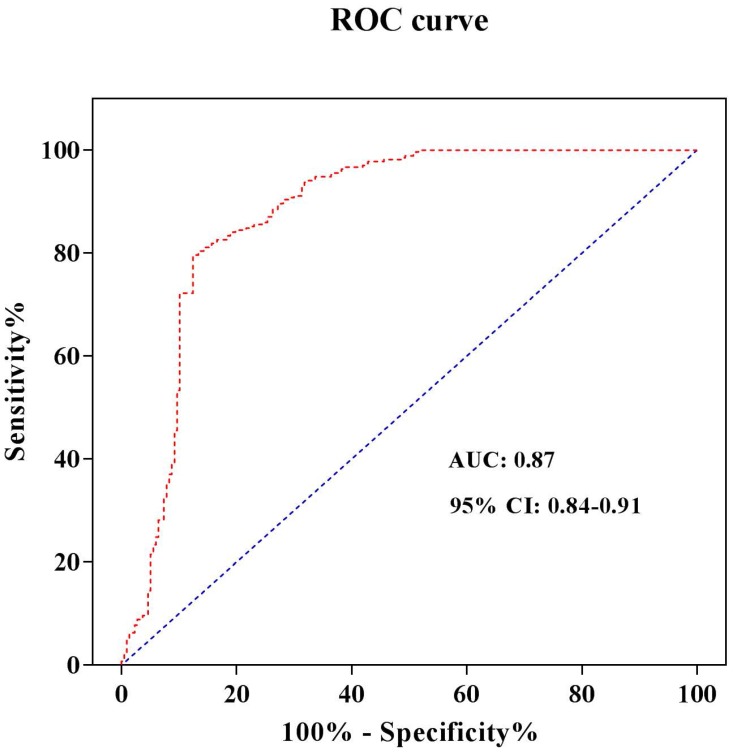
ROC curve analysis to determine the cut-off value to diagnose STEMI. Abbreviations: ROC curve, receiver operating characteristic curve; AUC, area under the curve; CI, confidence interval.

In order to refine the roles of different S100A1 levels in STEMI, we furthermore identify the S100A1 cut-off value. The serum hs-cTnT concentration was used as a diagnostic test for STEMI patients. STEMI was set to 1 and non-STEMI was set to 0. The receiver operating characteristic (ROC) curve was drawn with sensitivity as the ordinate and 1-Specificity as the abscissa. The area under the curve (AUC) was 0.87, and the 95% confidence interval was 0.84-0.91 (Figure 2). Both the sensitivity and specificity of S100A1 were higher when 1965.9 pg/mL was used as the threshold, and were 79.6% and 87.6%, respectively. Therefore, the patients were divided into two groups when we analyzed risks of different S100A1 levels on STEMI and conducted the cardiac function comparison based on this threshold: S100A1 ≥ 1965.9 pg/mL (n = 242) group and S100A1 < 1965.9 pg/mL (n = 245) group. The crude and adjusted risks of different S100A1 levels for STEMI in the studied population were shown in Table 4. OR of S100A1 > 1965.9 pg/mL to STEMI was 4.025 (95%CI: 1.735-9.260; P < 0.001) compared with that of S100A1 ≤ 1965.9 pg/mL. The results were similar (OR: 6.925; 95% CI: 4.15-11.375; P < 0.001) when we conducted the analysis after adjusted for age, gender, hypertension, diabetes mellitus, smoking habit, hs-cTnT, CK-MB, hs-CRP, NT-proBNP, and BMI.

### S100A1 is potentially associated with cardiac function in STEMI patients

**Figure 3:**
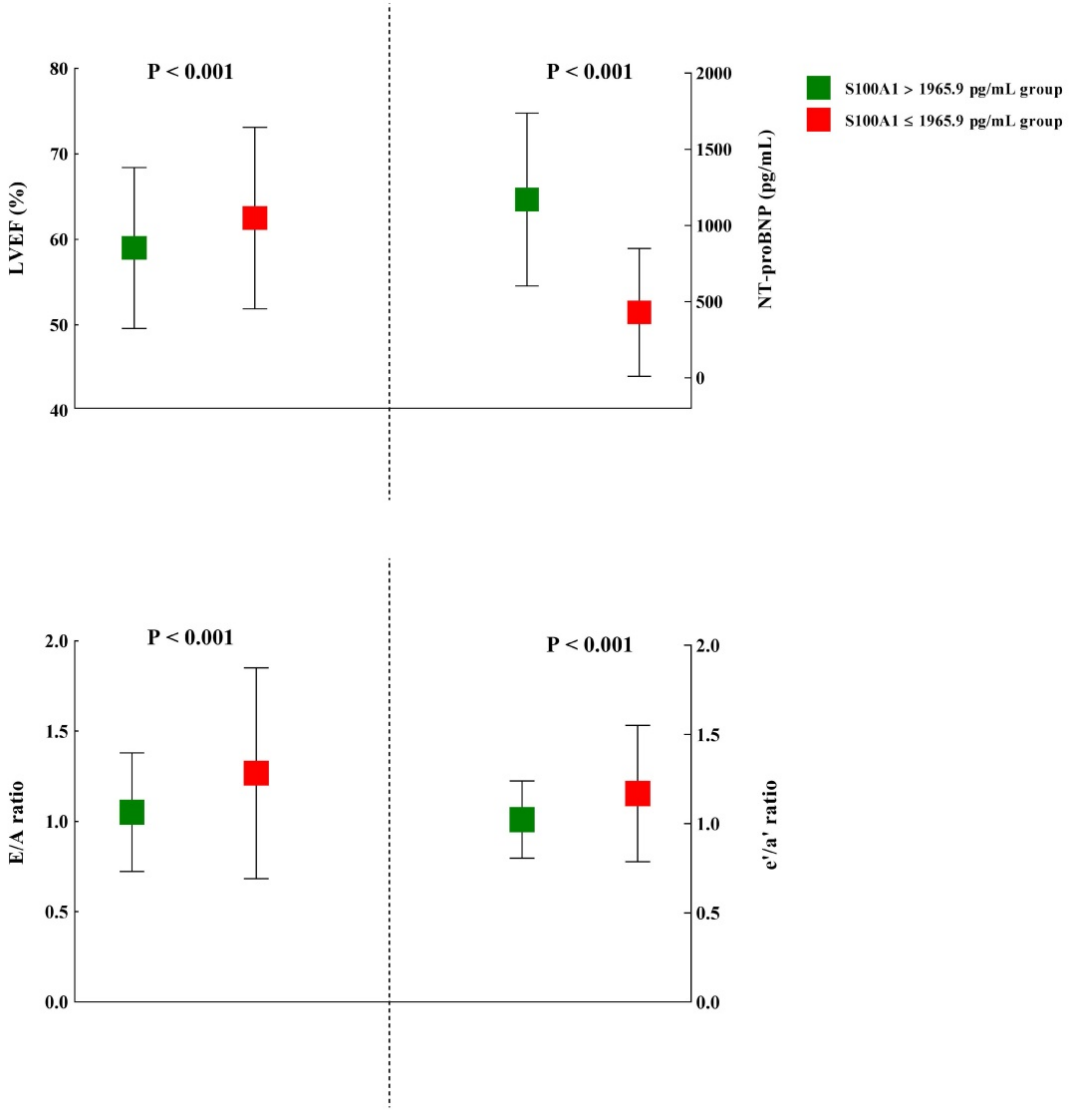
The comparison of several representative indexes of cardiac function between patients with higher (> 1965.9 pg/mL) and lower S100A1 levels (≤ 1965.9 pg/mL). Abbreviations: LVEF, left ventricular ejection fraction; NT-proBNP: N-terminal prohormone of brain natriuretic peptide; Peak E, the peak of early diastolic velocities; Peak A, the peak of late diastolic velocities; e', early diastolic mitral annulus velocities; a', late diastolic mitral annulus velocities.

The plasma S100A1 concentration was also found to be significantly inversely correlated with LVEF (r = -0.12; P = 0.01). Moreover, in STEMI and Control groups, we both detected significant correlation between LVEF and S100A1 level (STEMI group: r = -0.48, P = 0.01; Control group: r = -0.15, P = 0.03), respectively. The cardiac echocardiography results of the study subjects were shown in Table 5. Among the myocardial parameters, whether these were conventionally or DTI-derived, no statistical significance was detected in thickness of posterior wall (PWT), left ventricular end-systolic dimension (LVDs), Peak E, DT, s', and IVRT between S100A1 ≥ 1965.9 pg/mL and S100A1 < 1965.9 pg/mL groups. However, LVM, thickness of interventricular septum (IVS), and left atrial dimension (LAD) were significant higher in S100A1 ≥ 1965.9 pg/mL group, compared with S100A1 < 1965.9 pg/mL group (P = 0.006, P = 0.009, and P = 0.003, respectively). Most indicators of cardiac function showed statistical significance between these two groups. The NT-proBNP in S100A1 ≥ 1965.9 pg/mL group (1170.9 ± 567.2 pg/mL) was significantly higher than in S100A1 < 1965.9 pg/mL group (429.4 ± 419.4 pg/mL) (P < 0.001). The mean LVEF in S100A1 ≥ 1965.9 pg/mL group (59.0 ± 9.4%) was significantly lower than that of S100A1 < 1965.9 pg/mL group (62.5 ± 10.6%) (P < 0.001), which demonstrated a better systolic function in S100A1 < 1965.9 pg/mL group. The indexes of diastolic function such as E/A ratio, e'/a' ratio, and E/e' ratio, showed significantly better cardiac function in S100A1 < 1965.9 pg/mL group (all P < 0.001; Figure 3).

## Discussion

STEMI as a serious cardiovascular disorder is now a huge threat to human health with high morbidity and mortality. The early diagnosis and prompt reperfusion therapy are the effective measures that improve the clinical outcomes.

Thus, multiple factors influencing the diagnosis and outcomes should be taken into consideration and comprehensive therapeutic methods should also be established. Recent advances in underlying mechanisms of acute coronary syndrome have emphasized the importance of plasma biomarkers in diagnosis, risk stratification, therapeutic strategy and assessment of clinical outcomes 30. S100A1, the most abundant S100 isoform in cardiomyocytes, has attracted interest in cardiovascular disease since it may possess similarity of S100 family function that could regulate calcium homeostasis as calcium-binding proteins and thus potentially determined the cardiac function through calcium cycling 31-34. The present study preliminarily investigated the diagnostic significance of S100A1 in STEMI. Our data demonstrated that the plasma S100A1 level was significantly increased in STEMI patients. Even if adjusting for other cardiac risk factors, S100A1was statistically associated with the occurrence of STEMI and elevated S100A1 may be an independent risk factor for STEMI. Also interestingly, the findings indicated S100A1 level was positively correlated with CK-MB, hs-cTnT, and hs-CRP to a statistically significant extent, suggesting S100A1 may reflect the cardiac injury and inflammatory state of STEMI and could be used as a biomarker. In addition, we found S100A1 was significantly correlated with cardiac function parameters such as NT-proBNP and LVEF, which indicated S100A1 was closely associated with cardiac function, especially in the acute phase of STEMI.

An increasing number of studies support the notion that elevated S100A1 level may constitute an independent risk factor for the incidence of AMI. Usui A et al. 35 observed AMI patients have a higher S100A1 level than healthy adults, but the S100A1 level of angina pectoris patients was not significantly higher than the healthy subjects. Kiewitz R et al. 36 detected S100A1 level in varying conditions of ischemic heart disease and described the concentration-time course of S100A1 after acute cardiac ischemia. The results indicated that S100A1 was significantly increased after the occurrence of AMI, but the peak S100A1 value may vary as patients with different complications. Bi et al. 37 have established AMI rat model to study whether the S100A1 level can be used to diagnose acute myocardial ischemia, and they found that the longer duration of myocardial ischemia, the higher was the level of S100A1 in the early stage. In our study, we only included the STEMI patients that may represent the most serious type of AMI, the results were consistent with the report of Rohde's 38, who also demonstrated a significantly increased S100A1 level than in healthy subjects.

Cardiomyocyte injury could increase permeability of the myocardial cell membrane and result in release of several cardiac proteins into circulation. Thus, the appearance of such proteins in the bloodstream could be recognized as the biomarkers of severe cardiac ischemia in AMI including cTnT, cTnI, and CK-MB 39. Previously studies 37, 38 have proved that S100A1 could be promptly released into bloodstream and significant depletion of S100A1 in fibrous tissue and ischemic areas were also observed. These studies also pointed out exclusive S100A1 endocytosis by cardiac fibroblasts adjacent to damaged cardiomyocytes, followed by Toll-like receptor 4 (TLR4)-dependent activation of MAP kinases and NF-κB. These findings indicated S100A1 exerted an immunomodulatory and antifibrotic role and could beneficially modulate myocardial wound healing. Similar results were also reported by Yu et al. 40, which demonstrate that S100A1 can regulate the inflammatory response and oxidative stress in cardiomyocytes via TLR4/ROS/NF-κB pathway. Our data also support a role for S100A1 as an inflammatory indicator during acute phage in AMI. Plasma S100A1 was correlated with levels of hs-CRP, especially in patients with an elevated hs-CRP level (> 3mg/L), which is a relative higher risk for cardiovascular diseases. CRP is considered to be an important prognostic inflammatory factor in reflecting the activity and severity of atherosclerotic disease 41, 42. Plasma S100A1 is also correlated with CK-MB and cTnT in the study population. CK-MB and cTnT are both direct biomarkers of myocardial necrosis, which are potentially associated with CRP. The myocardial damage following acute coronary insufficiency also induces inflammatory response involve in elevated plasma CRP 43. These results demonstrated that plasma S100A1 may be an indicator of inflammation response as well as ischemic injury of myocardium in the acute phage of cardiac ischemic injury.

Cardiac dysfunction is another conventional cardiovascular risk associated with significant mortality, morbidity and health care expenditure 44. It has been long recognized that S100A1 levels was closely related with cardiac function since evidence showed that S100A1 mRNA and protein levels were diminished in failing cardiomyocytes and maintenance of normal cardiac function required more than 50% of normal S100A1 protein levels 45, 46. Moreover, in incubation with Rh-S100A1 seemed to promote cardiomyocyte survival due to endocytosis of this protein into cell and thus play a cardiac rescuing role 47. It is also proposed that S100A1 could maintain normal adult gene expression in myocardial tissue to inhibit cardiac hypertrophy. Decreased intracellular S100A1 in heart failure may unblock a fetal genetic program which initiated a hypertrophic response in damaged cardiomyocytes 48. However, previous studies mainly focused on the intracellular and tissue protein expression of S100A1, few studies revealed the changes in plasma S100A1 level in patients with post-infarction cardiac dysfunction. Our findings provide new evidence linking elevated plasma S100A1 levels with acute cardiac functional decline following STEMI as our findings showed significant correlation between plasma S100A1 and indicators of cardiac function including LVEF and pro-BNP. In addition, we detected a relative hypertrophic cardiac structure in patients with a higher plasma S100A1 level. And both systolic and diastolic cardiac function presented a significant decrease among these patients. Although the cardiac hypertrophy is a chronic process and acute release of S100A1 into bloodstream may not reflect the precise influence of plasma S100A1 level on myocardial remodeling, the results in our study still demonstrated S100A1 was a reasonable indicator of post-infarction cardiac function.

This study has several limitations. This study is a case-control clinical research; we did not analyze the prognostic value of S100A1 in STEMI. Due to the comprehensive laboratory protocol, we only included limited number of patients, thus some results may not be conclusive. Moreover, we only described statistical associations rather than the underlying mechanism of various parameter interactions.

## Conclusions

In summary, we found elevated S100A1 plasma level in STEMI patients and that S100A1 level was a likely complementary factor in combination with several markers to evaluate cardiac function in STEMI patients. Elevated plasma S100A1 level could be a useful predictor for STEMI and potentially reflect the myocardium injury and inflammation response during the acute coronary ischemia. What remains unclear is the exact mechanism by which elevated plasma S100A1 level is associated with STEMI. The precise role of S100A1 in the pathogenesis of STEMI still requires further investigations.

## Figures and Tables

**Table 1:** Baseline characteristics of patients in two groups

	STEMI group (n=270)	Control group (n=217)	P value
Sex (M/F)	161/109	127/90	0.439
Age (yrs)	66.9±9.4	68.0±9.7	0.194
Smoking history (n, %)	117 (43.3%)	89 (41.0%)	0.336
Hypertension (n, %)	108 (40.0%)	87 (40.1%)	0.529
Diabetes mellitus (n, %)	96 (35.6%)	40 (18.4%)	< 0.001
hs-cTnT (ng/mL)	1.183±0.996	0.007±0.002	< 0.001
CK-MB (U/L)	96.9±71.6	13.7±4.8	< 0.001
hs-CRP (mg/dL)	6.3±2.0	2.8±1.6	< 0.001
NT-proBNP (pg/mL)	1212.4±532.5	282.2±180.2	< 0.001
LVEF (%)	58.3±9.4	63.8±10.3	< 0.001
FBG (mmol/L)	5.1±2.4	4.2±2.0	< 0.001
HbA1C (%)	5.9±3.5	5.1±3.2	0.008
TC (mmol/L)	4.6±0.9	4.5±0.9	0.335
TG (mmol/L)	2.0±0.4	1.9±0.4	0.212
HDL-C (mmol/L)	1.3±0.8	1.2±0.7	0.054
LDL-C (mmol/L)	3.2±1.9	2.9±1.8	0.072
BMI (kg/m^2^)	25.3±3.1	25.0±3.2	0.275
S100A1 (pg/mL)	3197.7±1576.0	1423.5±1315.5	< 0.001

hs-cTnT: hypersensitive cardiac troponin T; CK-MB, creatine kinase MB; hs-CRP: hypersensitive C-reactive protein; NT-proBNP: N-terminal prohormone of brain natriuretic peptide; LVEF, left ventricular eject fraction; FBG: fasting blood glucose; HbA1C: hemoglobin A1c; TC: total cholesterol; TG: triglyceride; HDL-C: high density lipoprotein-cholesterol; LDL-C: low density lipoprotein-cholesterol; BMI: body mass index.

**Table 2:** The S100A1 levels among patients with or without cardiovascular risk factors

	Plasma S100A1 level (pg/mL, mean ± SD)	P value
**Male**	2438.3 ± 1781.0	0.63
**Female**	2362.0 ± 1604.2	
**Age<65 yrs**	2314.2 ± 1772.7	0.19
**Age≥65 yrs**	2519.0 ± 1627.6	
**With hypertension**	2599.9 ± 1893.1	0.04
**Without hypertension**	2278.4 ± 1565.6	
**With diabetes**	2626.1 ± 1623.2	0.08
**Without diabetes**	2322.3 ± 1736.9	
**Smokers**	2614.7 ± 1829.8	0.02
**Non-smokers**	2255.0 ± 1602.3	

**Table 3:** Multivariate logistic regression for identification of independent predictors of STEMI

Variables	OR	95% CI	P value
Age	0.959	0.916-1.003	0.068
Gender	1.166	0.445-3.056	0.755
Hypertension	1.004	0.697-1.446	0.984
Diabetes Mellitus	2.439	1.597-3.731	< 0.001
Smoking history	1.100	0.766-1.580	0.607
hs-cTnT	0.308	0.209-0.455	< 0.001
CK-MB	0.628	0.563-0.701	< 0.001
hs-CRP	0.338	0.279-0.410	< 0.001
NT-proBNP	0.456	0.275-0.756	0.002
S100A1	0.671	0.500-0.891	< 0.001
BMI	1.033	0.975-1.094	0.275

STEMI, ST-segment elevation myocardial infarction; hs-cTnT: hypersensitive cardiac troponin T; CK-MB, creatine kinase MB; hs-CRP: hypersensitive C-reactive protein; NT-proBNP: N-terminal prohormone of brain natriuretic peptide; BMI: body mass index; OR, odds ratio; CI, confidence interval.

**Table 4:** Relative risks for STEMI according to the serum S100A1 levels

	Univariate				Multivariate^#^		
	**OR**	**95% CI**	**P value**		**OR**	**95% CI**	**P value**
S100A1 > 1965.9 pg/mL	4.025	1.735-9.260	< 0.001		6.925	4.15-11.375	< 0.001
S100A1 ≤ 1965.9 pg/mL	Reference				Reference		

# Adjusted for age, gender, hypertension, diabetes mellitus, smoking habit, hs-cTnT, CK-MB, hs-CRP, NT-proBNP, and BMI. STEMI, ST-segment elevation myocardial infarction; OR, odds ratio; CI, confidence interval.

**Table 5:** Cardiac structure and function in the study population

	S100A1 > 1965.9 pg/mL group (n=242)	S100A1 ≤ 1965.9 pg/mL group (n =245)	P value
**Cardiac structure**			
LVM (g)	147.5 ± 26.1	139.5 ± 36.1	0.006
IVS (cm)	0.93 ± 0.18	0.89 ± 0.15	0.009
PWT (cm)	0.87 ± 0.12	0.86 ± 0.15	0.233
LAD (cm)	3.69 ± 5.55	3.54 ± 5.70	0.003
LVDd (cm)	4.99 ± 0.55	4.90 ± 0.51	0.071
LVDs (cm)	3.02 ± 0.52	3.09 ± 0.57	0.167
**Cardiac function**			
NT-proBNP (pg/mL)	1170.9 ± 567.2	429.4 ± 419.4	< 0.001
LVEF (%)	59.0 ± 9.4	62.5 ± 10.6	< 0.001
Peak E (cm/s)	76.2 ± 17.6	75.1 ± 28.0	0.605
Peak A (cm/s)	77.6 ± 15.9	67.5 ± 22.6	< 0.001
DT (s)	2.10 ± 0.22	2.07 ± 0.22	0.085
IVRT (s)	0.10 ± 0.08	0.09 ± 0.09	0.644
s' (cm/s)	7.47 ± 1.33	7.61 ± 1.34	0.261
e' (cm/s)	7.15 ± 1.53	7.92 ± 2.56	< 0.001
a' (cm/s)	7.17 ± 0.35	7.08 ± 0.38	0.004
E/A ratio	1.05 ± 0.33	1.27 ± 0.58	< 0.001
e'/a' ratio	1.02 ± 0.22	1.17 ± 0.38	< 0.001
E/e' ratio	10.9 ± 1.23	9.70 ± 2.02	< 0.001

Abbreviations: LVM, left ventricular mass; IVS, thickness of interventricular septum; PWT, thickness of posterior wall of left ventricle; LAD, left atrial dimension; LVDd, left ventricular end-diastolic dimension; LVDs, left ventricular end-systolic dimension; NT-proBNP: N-terminal prohormone of brain natriuretic peptide; LVEF, left ventricular ejection fraction; Peak E, the peak of early diastolic velocities; Peak A, the peak of late diastolic velocities; DT, the deceleration time of the peak E velocity; IVRT, isovolumic relaxation time; s', systolic motion wave velocities; e', early diastolic mitral annulus velocities; a', late diastolic mitral annulus velocities.

## Abbreviations

CAD: coronary artery disease
STEMI: ST-elevation myocardial infarction
cTn: cardiac troponin
hs-cTn: high-sensitivity cardiac troponin
CK-MB: creatine kinase MB
MYO: myoglobin
NT-proBNP: N-terminal prohormone of brain natriuretic peptide
CRP: C-reactive protein
PCI: percutaneous coronary intervention
FBG: fasting blood glucose
HbA1C: hemoglobin A1c
TC: total cholesterol
TG: triglycerides
HDL: high density lipoprotein
LDL: low density lipoprotein
hs-cTnT: high sensitive cardiac troponin T
CAG: coronary angiography
LVM: Left ventricular mass
LVEF: left ventricular ejection fraction
DT: deceleration time
IVRT: isovolumic relaxation time
DTI: Doppler tissue imaging
ELISA: enzyme-linked immunosorbent assay
ROC: receiver operating characteristic
AUC: area under the curve
PWT: thickness of posterior wall
LVDs: left ventricular end-systolic dimension
IVS: thickness of interventricular septum
LAD: left atrial dimension
Peak E: the peak of early diastolic velocities
Peak A: the peak of late diastolic velocities
e': early diastolic mitral annulus velocities
a': late diastolic mitral annulus velocities
OR: odds ratio
CI: confidence interval
TLR4: Toll-like receptor 4
